# Effect of Gas Annealing on the Electrical Properties of Ni/AlN/SiC

**DOI:** 10.3390/mi12030283

**Published:** 2021-03-08

**Authors:** Dong-Hyeon Kim, Michael A. Schweitz, Sang-Mo Koo

**Affiliations:** Department of Electronic Materials Engineering, Kwangwoon University, 20 Kwangwoon-ro, Nowon-gu, Seoul 01897, Korea; gogomatt@kw.ac.kr (D.-H.K.); michael.schweitz@schweitzlee.com (M.A.S.)

**Keywords:** aluminum nitride, silicon carbide, Schottky barrier diodes, radio frequency sputtering, X-ray diffraction, X-ray photoelectron spectroscopy

## Abstract

It is shown in this work that annealing of Schottky barrier diodes (SBDs) in the form of Ni/AlN/SiC heterojunction devices in an atmosphere of nitrogen and oxygen leads to a significant improvement in the electrical properties of the structures. Compared to the non-annealed device, the on/off ratio of the annealed SBD devices increased by approximately 100 times. The ideality factor, derived from the current-voltage (IV) characterization, decreased by a factor of ~5.1 after annealing, whereas the barrier height increased from ~0.52 to 0.71 eV. The bonding structure of the AlN layer was characterized by X-ray photoelectron spectroscopy. Examination of the N 1 s and O 1 s peaks provided direct indication of the most prevalent chemical bonding states of the elements.

## 1. Introduction

Wide bandgap semiconductor materials have superior thermal and electrical properties compared to those of conventional semiconductors, and thus have potential for use in high-power, high-temperature, microwave and optoelectronic applications [1,2,3]. Next-generation high-temperature electronics will require increasing use of semiconductor materials such as SiC, GaN, AlN, and AlGaN due to their superior electrical properties resulting from their wide band–gap and high thermal conductivity. AlN has the largest bandgap of 6.2 eV and critical electric field of 11.7 MV/cm, as well as the highest thermal conductivity of 320 W/mK among group III materials [4,5]. These material properties make AlN highly versatile for use in high-power and high-temperature applications, including motor drives, energy conversion systems, high-temperature sensors, and space exploration. AlN films can be manufactured by several different processes, including chemical vapor deposition, molecular beam epitaxy, and radio frequency (RF)-sputtering. Compared to high-temperature thin film deposition techniques, RF-sputtering is cheaper and simpler to implement. Importantly, RF-sputtering offers the possibility to manufacture high-quality, large-scale films with desirable material properties, including on temperature-sensitive substrates if required [6,7,8]. However, RF-sputtering results in a low polar field which reduces the performance of high electron mobility transistors [9]. Furthermore, AlN thin films grown by RF-sputtering contain defects related to oxygen impurities, resulting in impaired electrical and optical properties. The theoretical modeling of oxygen in semiconductor materials remains computationally challenging, largely because traditional empirical or semi-empirical methods fail to satisfactorily explain the large electronegativity of oxygen, along with its chemical binding properties.

Annealing is a potentially important process for manufacturing high-quality compound semiconductors with thin films of materials such as GaAs, SiC, and AlN because the process appears to have the capacity to reduce unintentional defects in these films by orders of magnitude [10,11,12]. However, previous experimental studies have focused mainly on the sputtering parameters, such as pressure, power, sputtering ambient, and target distance [13,14]. It is known that annealing improves the crystallization of films similar to those mentioned [15]; however, very few researchers have studied the influence of post-annealing treatment on the surface morphology and crystallization orientation of AlN films [16].

While the properties of AlN films are determined by the deposition method, they are also affected by post-deposition treatment parameters, such as annealing temperature and duration [17]. The manufactured SBDs are expected to be of use in high temperature applications. Their behavior and use as temperature sensors for temperatures up to 475 K is explored in [18]. Further investigations of the manufactured device properties are ongoing. Indicative values of, for instance, breakdown voltage of related AlN thin film structures can be found in [19]. In this study, we investigate the influence of post-deposition annealing in nitrogen (N_2_) and oxygen (O_2_) atmospheres on the electrical properties of AlN thin films. We demonstrate that gas annealing of AlN thin films in either a nitrogen or oxygen atmosphere results in films with lower leakage currents at 300 K than for non-annealed AlN thin films. This effect was demonstrated by characterizing the electrical properties of manufactured heterojunction (Ni/AlN/SiC) Schottky barrier diodes (SBDs).

## 2. Materials and Methods

**Figure 1:**
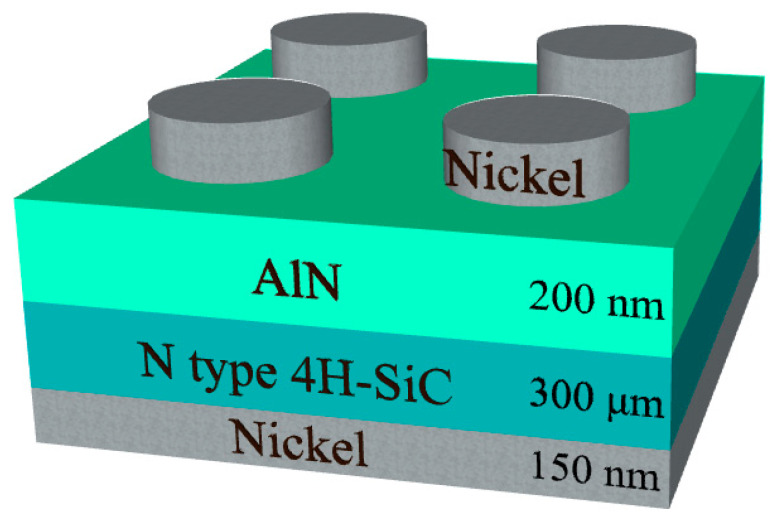
AlN/4H-SiC heterojunction diode structure.

A schematic of the manufactured vertical AlN Schottky barrier diode (SBD) is shown in Figure 1. N-type 4H-SiC wafers (base substrate: N_D_ = 1 × 10^19^ cm^−3^; n-type epitaxial layer: N_D_ = 5 × 10^16^ cm^−3^) acted as starting substrates. The SiC substrate was cleaned in a sulfuric peroxide mixture (sulfuric acid (H_2_SO_4_) to hydrogen peroxide (H_2_O_2_) ratio of 4:1), after which the native SiO_2_ layer was stripped using a buffered oxide etch (BOE) solution. A 150 nm thick Ni-film was then deposited by e-beam evaporation to create a diode cathode on the reverse side of the substrate. The samples were subjected to rapid thermal annealing (RTA) at 1323 K in N_2_ for 90 s to form nickel silicide (Ni_2_Si) ohmic contacts. AlN films were subsequently deposited by RF sputtering of a 99.9% pure AlN target onto the substrate at 300 K under injection of high purity argon gas (99.999%) with a flow rate of 5.5 sccm, using a mass flow controller. The sputtering power was 150 W, and the target diameter was 5.08 cm. Deposition chamber working pressure was maintained at 10 mTorr during the 120 min deposition step, resulting in a film thickness of approximately 200 nm. An Alpha-Step stylus profilometer and atomic force microscopy (AFM) were used to measure the thickness of the AlN films. AFM thickness measurements are in good agreement with measurements performed by ellipsometry [20]. AlN thin film samples either remained as deposited, i.e., not annealed, or were annealed at 773 K for 30 min, in either an ambient nitrogen or oxygen atmosphere. Top electrode contacts were created by depositing a 150 nm thick nickel layer on the AlN thin films, thus completing the sample SBDs. The mobility of the AlN films were measured using a Ecopia HMS-5000 Hall Effect Measurement System. The four Hall measurement probes were each connected through ohmic contacts located at each upper corner of the AlN film samples.

Before deposition of the top electrodes, the samples were subjected to X-ray diffraction (XRD) measurement, X-ray photoelectron spectroscopy (XPS), and Fourier transform infrared spectroscopy (FTIR). XPS was performed to analyze the orientation of the wurtzite crystal structure of the AlN thin films [21]. Annealing atmosphere influence on the AlN film binding energies was measured by X-ray photoelectron spectroscopy (XPS). Al, N, and O contents of the films were estimated from Al 2p, N s, and O s XPS core-level spectra. The AlN film samples were analyzed by Fourier transform infrared spectroscopy (FTIR), and, finally, the finished SBDs were characterized by current voltage (I-V) measurements at 300 K using a semiconductor analyzer (Keithley 4200-SCS, Tektronix, Beaverton, OR 97077, USA).

## 3. Result and Discussion

**Figure 2:**
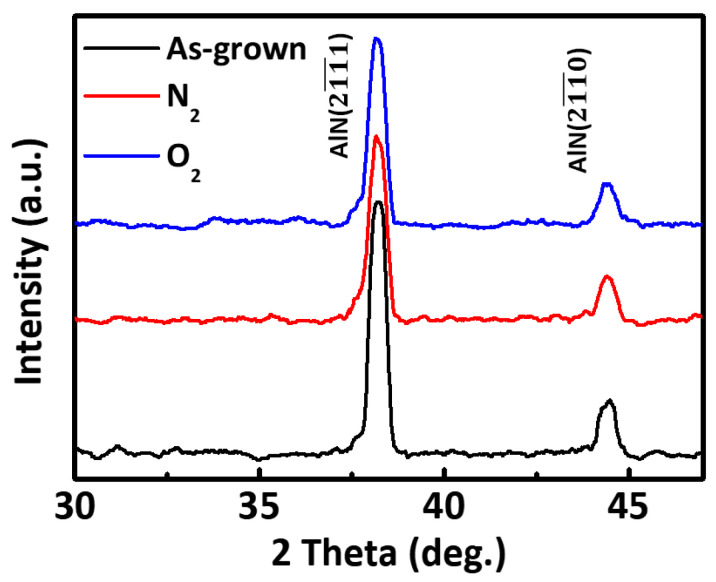
XRD patterns of the as-grown, N_2_, and O_2_ annealed AlN films.

Figure 2 shows the XRD patterns of the untreated (“as-grown”, i.e., before annealing) and gas (nitrogen or oxygen) annealed AlN films, indicating the main crystal plane orientations of the films. The three graphs in Figure 2 each display two intensity peaks at 2θ = 38.2° and 44.4°, corresponding to the ($2\bar{11}1$) and ($2\bar{11}0$) AlN crystal diffraction planes, respectively. The amplitudes of these peaks decrease after annealing. The different XRD patterns indicate that the samples annealed in either N_2_ or O_2_ atmospheres were influenced at the crystal domain level to cause the observed decrease in intensity and full width at half maximum (FWHM) [22].

**Figure 3:**
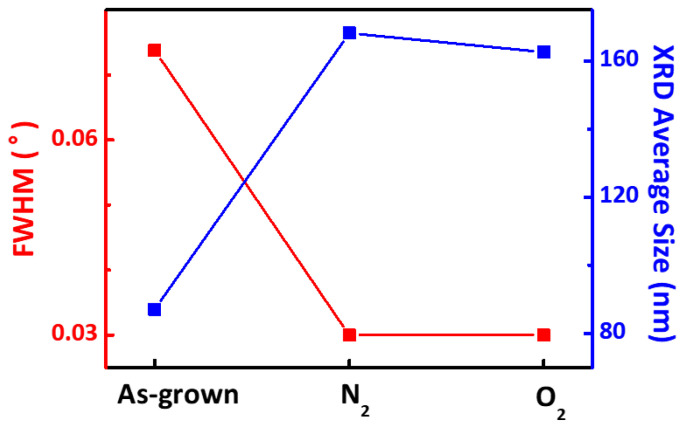
Full width at half maximum (FWHM) of the XRD Al$\left(2\bar{11}0\right)$ peak and the corresponding average crystal size for the as-grown, N_2_, and O_2_ annealed AlN films.

Figure 3 shows the FWHM of the Al ($2\bar{11}0$) peak and the corresponding average AlN crystal domain size. The average domain size of the as-grown, N_2_ annealed, and O_2_ annealed samples was calculated according to the Scherrer equation given by

(1) $$D=\;\frac{Κ\lambda }{\beta \sin\left(\theta \right)}$$

where K is the shape factor (0.9), *λ* is the X-ray wavelength (1.5406 Å), *β* is the FWHM of the peaks in radians, and *Ɵ* is the Bragg diffraction angle. The results indicate that the FWHM was reduced as a result of the annealing process. The average grain sizes of the as-grown, N_2_, and O_2_ annealed samples estimated by the Scherrer equation and the XRD data are 87.07 nm, 168.17 nm, and 162.64 nm, respectively. This indicates increases in both uniformity and the level of crystallinity [16]. This result suggests that annealing can to some degree remove or “soften” the grain boundaries in the film. Consequently, the annealed samples contain fewer or less distinct grain boundaries, leading to a reduced number of charge carrier traps or scattering obstacles along grain boundaries [23].

**Figure 4:**
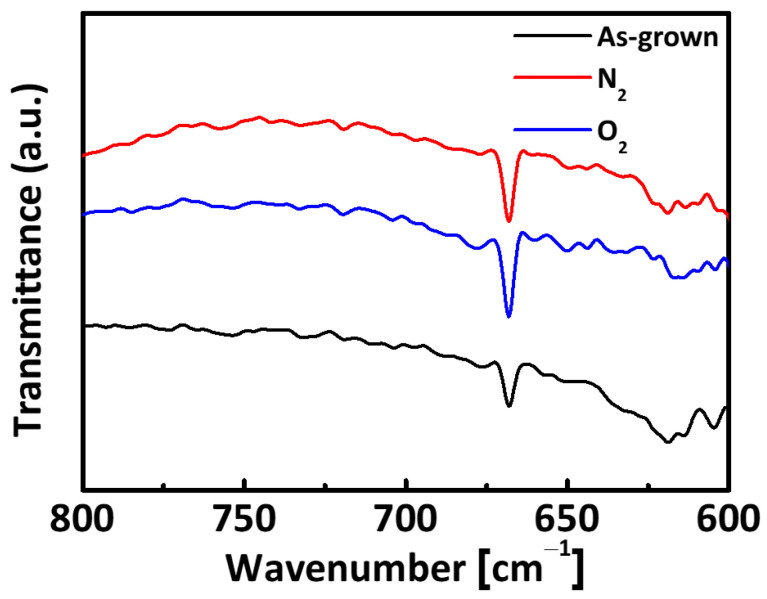
FTIR transmittance spectra of the as-grown, N_2_, and O_2_ annealed AlN films.

Figure 4 shows the FTIR spectra of AlN films before and after N_2_ and O_2_ annealing. Spectra were obtained in the range from 600 to 800 cm^−1^. The absorption peak at 668 cm^−1^ corresponds to the characteristic value of aluminum nitride. The magnitude of this peak increased with gas annealing, as seen in Figure 4 [24]. Furthermore, the FTIR spectra in Figure 4 show increased overall transmittance of the N_2_ and O_2_ annealed AlN films, indicating improved film crystallinity [25].

**Figure 5:**
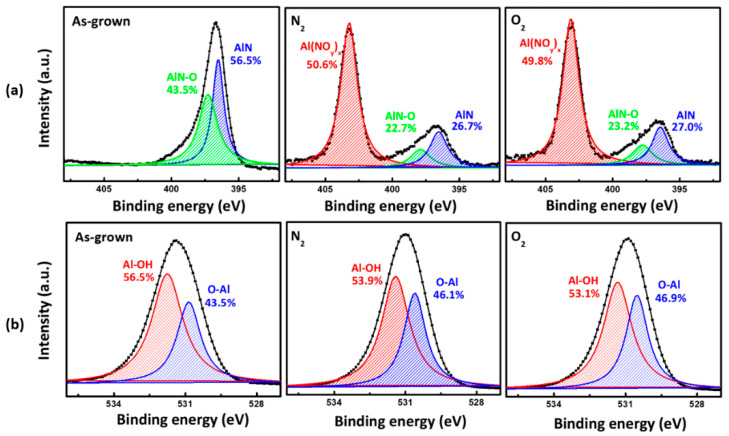
XPS spectra (**a**) (N 1 s) and (**b**) (O 1 s) of the as-grown, N_2_, and O_2_ annealed AlN films.

In order to investigate the effect of the annealing process on the chemical bonding state of nitrogen atoms in the AlN layer, we performed an XPS core level measurement. Figure 5a,b show the N 1 s and O 1 s core level XPS spectra for the AlN films. Figure 5a shows these spectra were deconvoluted into three components with peaks at 403.2 ± 0.1, 397.4 ± 0.2, and 396.7 eV, which correspond to the chemical bonding states of Al-(NO_x_)_y_, AlN-O, and Al-N, respectively, as previously reported in the literature [26]. The XPS response after annealing displays a new peak at 403.2 ± 0.1 eV, distinct from the expected one at 397.4 ± 0.2 eV [27]. This indicates increased levels of surface oxygen, which is understood to result from the creation of an oxide film.

**Figure 6:**
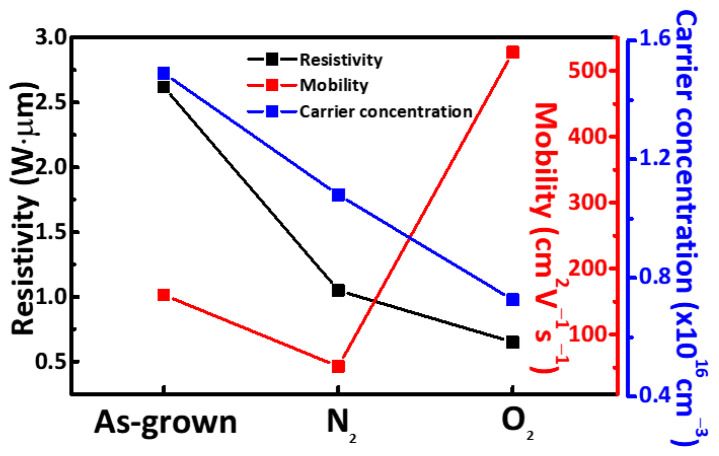
Mobility and carrier concentration of the as-grown, N_2_, and O_2_ annealed AlN films.

When it reducing the relative nitrogen content, AlN chemical bonds break up. This is likely due to the result of the oxidation of the AlN surface barrier that is presumed to occur [28]; also, annealing has related to the creation of nitrogen defects in the form of interstitial N_2_ trapped between the surface oxide film and AlN film interlayer. Figure 5b shows the measured XPS core-level O 1s spectra. These can be deconvoluted into two peaks at 531.78 and 530.88 eV of the as-grown sample, corresponding to the Al-OH and O-Al chemical bond states, respectively [29]. After gas annealing, in the N_2_ annealed sample, Al–OH and O–Al binding energies shifted to 531.38 eV and 530.58 eV, respectively. Said energies were 0.4 eV and 0.3 eV lower than those of the as-grown sample. In the O_2_ annealed sample, the Al–OH and O–Al binding energies shifted to 531.32 eV and 530.50 eV, which in turn were lower than the respective binding energies of the N_2_ annealed sample. Additionally, the relative area of the Al–OH peak decreased. Annealing effectively decomposes the OH- species in the AlN film. Simultaneously, the residual OH- bonds release Al atoms to form more Al-O bonds. Then, the OH- groups are removed and the oxygen vacancies are filled [30]. The relationship between the electrical properties of the AlN thin film samples and the film quality was investigated by Hall measurements. Figure 6 shows charge carrier mobility and concentration relative to the different gas annealing conditions. The maximum achieved carrier mobility (528 cm^2^/Vs) was observed in the O_2_ annealed sample. A reduced number of crystal defects result in higher mobility and decreased charge carrier concentration [31]. From the literature, we infer that also in AlN thin films, grain boundaries are the main source of defects that limit charge carrier mobility and give rise to charge carriers [32,33]. Film crystallinity is proportional to the average crystal grain size, which in turn is inversely proportional to the density of grain boundaries. With the O_2_ annealed device having a larger average grain size, it is reasonable to expect that the density of the grain boundaries may have decreased accordingly. This, in turn, would explain the observed reduction in carrier concentration and the increase in mobility.

**Figure 7:**
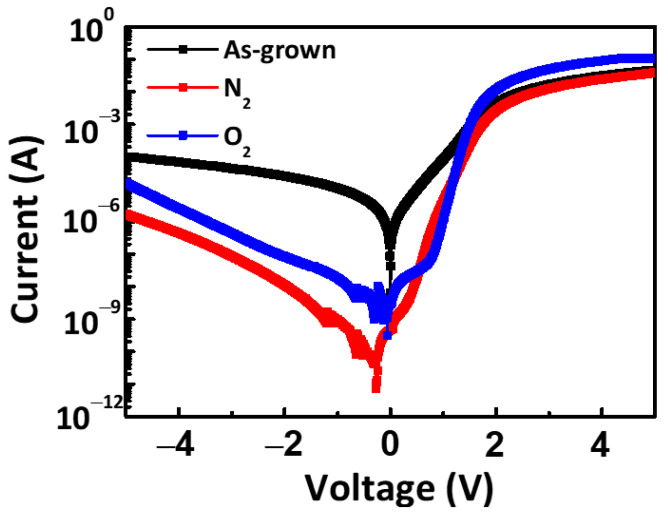
I–V characteristics for the as-grown, N_2_, and O_2_ annealed AlN/4H-SiC SBDs.

Figure 7 shows the typical I-V characteristics of the fabricated AlN/4H-SiC SBDs measured on a logarithmic scale. The diode currents were measured for terminal potentials ranging from −5 V to +5 V. From 0 V to 2 V, the forward current of the as-grown sample was higher than that of the annealed samples (N_2_ annealed, O_2_ annealed), while for voltages higher than 2 V, the forward current of the O_2_ annealed sample was the highest. For the case of reverse bias, the annealed AlN SBDs exhibited lower leakage currents (~1.3 × 10^−6^ A) than the as-grown AlN SBD (9.5 × 10^−5^ A).

**Figure 8:**
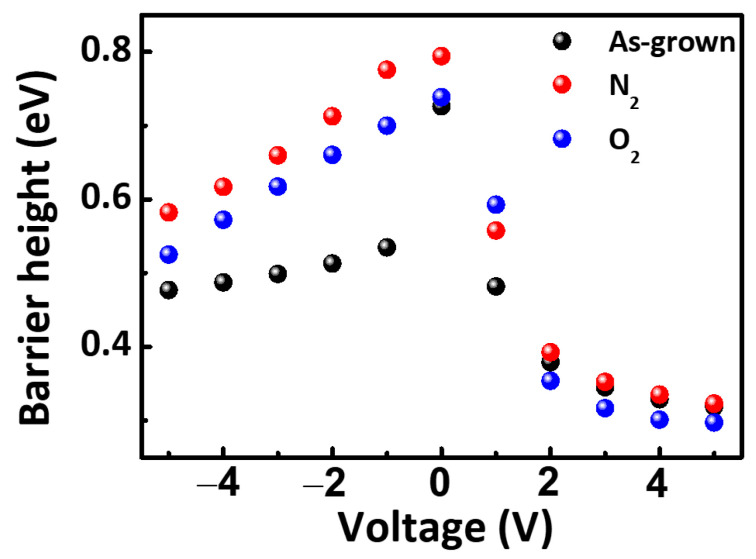
Barrier height of AlN on 4H-SiC SBDs with different gas annealing.

The Schottky barrier height ($\phi_{B}$) of the manufactured diodes was calculated according to Equation (2) and shown in Figure 8:

(2) $$I_{S}=AA^{*}T^{2}\left[\exp\left(\frac{-q\phi_{B}}{kT}\right)\right]$$

where $\phi_{B}$ is the barrier height, *A* is the effective area of the diode for current transport, *I_S_* is the saturation current, *T* is the measurement temperature, and $A^{*}$ is the Richardson constant (theoretically ~57.6 A cm^−1^ K^−2^ for AlN) [34,35]. The Figure 8 shows the N_2_ annealed device exhibited the highest Schottky barrier height $\phi_{B}$ = 0.71 eV at reverse bias. From 0 V to 2 V forward bias, the O_2_ annealed device had the highest barrier height $\phi_{B}$ = 0.59 eV, which can be explained by the filled oxygen vacancies identified from the Al-OH/Al-O peak ratio of the XPS O 1s data. After 2 V forward bias, the O_2_ annealed device also had the lowest barrier height $\phi_{B}$ = 0.29 eV, which can be explained by the Hall carrier mobility.

**Figure 9:**
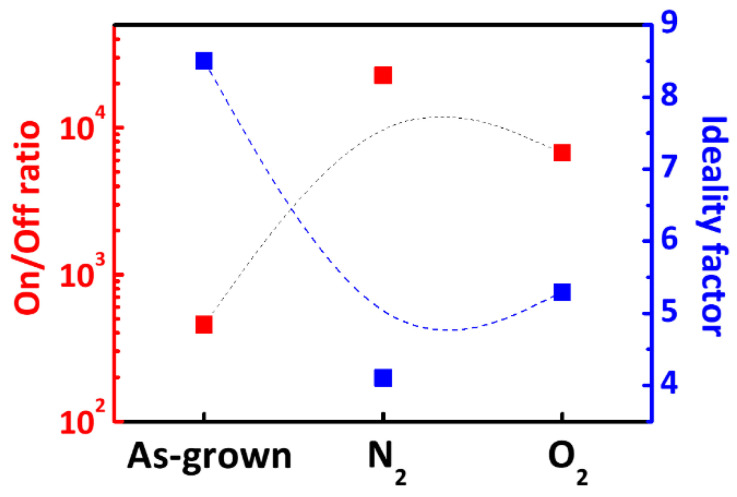
On/off ratio and ideality factor of AlN on 4H-SiC SBDs with different gas annealing.

The I_on_/I_off_ ratio and ideality factor values of the fabricated devices are shown in Figure 9. According to thermionic emission (TE) theory, the SBD I-V curves in forward bias can be expressed in the form of Equations (3)–(5) [36,37].

(3) $$\emptyset_{B}=\frac{kT}{-q}ln\left(\frac{I_{ST}}{AA^{*}T^{2}}\right)$$

(4) $$I=I_{s}\left[\exp\left(\frac{q\left(V-IR_{s}\right)}{\eta kT}\right)-1\right]$$

(5) $$\eta =\frac{q}{KT}\left[\frac{dV}{d\left(lnI\right)}\right]$$

where η is the ideality factor, *q* is the elementary electric charge, *k* is the Boltzmann constant, *T* is the absolute temperature, and *I_s_* is the saturation current. The ideality factor value is explained assuming a Gaussian distribution of the barrier height around the AlN/4H-SiC interface. As Figure 9 shows, the lower the ideality factor, the greater the barrier height. The on/off ratios at room temperature of the as grown, N_2_ annealed, and O_2_ annealed samples were calculated to be ~4.5 × 10^2^, ~2.2 × 10^4^, and ~6.7 × 10^3^, respectively. The corresponding ideality factors for the as grown, N_2_ annealed, and O_2_ annealed samples were 8.5, 4.1, and 5.29, respectively. The on/off ratio of the annealed device was two orders of magnitude (~100 times) higher than the on/off ratio of the as-grown device, while the ideality factor was the lowest (4.1) for the N_2_ annealed sample.

In summary, after the annealing process, the electrical conduction properties of the AlN SBDs improved.

## 4. Conclusions

The influence of different annealing atmospheres on AlN thin films was investigated. We measured and analyzed the electrical characteristics of an AlN SBD before and after the gas of nitrogen and oxygen annealing. For the N_2_ and O_2_ annealed sample, we observed that the AlN thin films were formed with relatively large grain sizes 168.17 nm and 162.62 nm, respectively, and higher magnitude of Al-N bond peaks. These less distinct grain boundaries related to a reduced number of charge carrier traps or scattering obstacles along grain boundaries. Charge carrier mobility was the highest (528 cm^2^/Vs) for the O_2_ annealed sample. From this sample, we also observed a relatively a reduced barrier height, and increased forward current. The high-temperature annealing process caused the decomposition of the Al-OH bonds; as a result, the relative area of the Al-O peak increased, while the number of oxygen vacancies decreased in O_2_ annealed sample. Consequently, the current decreased with the increasing electric resistance until 2 V. One main result of this study is that the characteristic XPS data of the N 1 s region show unusual feature at 404 eV. It is related that the reduced reverse leakage current is a result of the trapped nitrogen defect. The barrier height decreased with improved conductivity, which in turn resulted in an improved on/off ratio in the N_2_ annealed devices. In conclusion, the N_2_ annealed sample had the lowest reverse leakage current and the O_2_ annealed sample displayed the highest forward current level. Gas annealing thus constitutes a method for controlling the electrical properties of manufactured AlN thin films.

## Data Availability

Data is contained within the article.
